# Supplementary material for: Assessing the effects of disease-specific programs on health systems: An analysis of the Bangladesh Lymphatic Filariasis Elimination Program’s impacts on health service coverage and catastrophic health expenditure
Source: PLoS Negl Trop Dis. 2021 Nov 23;15(11):e0009894. doi: 10.1371/journal.pntd.0009894 (PMC8651132; doi:10.1371/journal.pntd.0009894)
Supplement: S1 File — (DOCX) [file pntd.0009894.s001.docx]

**Supplement 1. Calculating the composite coverage index**

The following definitions are from DHS Final Report 2014 [1]

Unmet need for family planning – “…fecund women who are not using contraception but who wish to postpone the next birth (spacing) or stop childbearing altogether (limiting). Specifically, women are considered to have unmet need for spacing if they are:

- At risk of becoming pregnant, not using contraception, and either do not want to become pregnant within the next two years or are unsure if or when they want to become pregnant.
- Pregnant with a mistimed pregnancy
- Postpartum amenorrhoeic for up to two years following a mistimed birth and not using contraception [p. 91].”

Antenatal care (ANC) by a skilled provider – “Information on ANC was assessed for women who gave birth in the three years preceding the survey. Among women with two or more live births during the three-year period, data refer to the most recent live birth only… Women were asked to report on all persons they saw for the ANC for their last birth. However, if a woman saw more than one provider, only the provider with the highest qualifications was considered in the tabulation of results [p. 112].”

Delivery assisted by a skilled health professional – “Women interviewed in the 2014 BDHS reported on the place and type of assistance during delivery of all children born in the three years before the survey [p. 118].”

Childhood vaccinations (BCG, Measles, DPT) – The 2014 BDHS collected data on childhood vaccinations for all surviving children born during the five-year period before the survey…If the mother was able to show the vaccination care, the dates of the vaccinations were transferred from the card to the survey questionnaire. If the vaccination card was not available, mothers were asked to recall weather the child had received each vaccination [p. 139].”

Treatment of Childhood Diarrhea – “For children with diarrhea in the two weeks before the survey, the mother was asked what she did to treat the diarrhea [p. 142]”

Treatment for cough with rapid breathing and fever – Respondents “were asked if their children under age 5 had experienced symptoms of [acute respiratory infection] in the two weeks preceding the survey [p. 147].” “In the 2014 BDHS, the interviewing teams were provided with a list of drug names to facilitate identifying whether the drug given to the child reported to have ARI symptoms is an antibiotic or not [p. 148].”

Data dictionary

1. NIPORT, Mitra and Associates, ICF International. Bangladesh Demographic and Health Survey 2014. Dhaka, Bangladesh, and Rockville, Maryland, USA: NIPORT, Mitra and Associates, and ICF International; 2016.
