# Supplementary material for: Assessing the effects of disease-specific programs on health systems: An analysis of the Bangladesh Lymphatic Filariasis Elimination Program’s impacts on health service coverage and catastrophic health expenditure
Source: PLoS Negl Trop Dis. 2021 Nov 23;15(11):e0009894. doi: 10.1371/journal.pntd.0009894 (PMC8651132; doi:10.1371/journal.pntd.0009894)
Supplement: S2 File — (DOCX) [file pntd.0009894.s002.docx]

**Supplement 2. Calculating incidence of catastrophic health expenditures**

The calculations included in this supplement are based on those provided in the “Users’ manual for handling resampled micro data of Bangladesh Household Income and Expenditure Survey” [1].

|  | **HIES 2000** | | **HIES 2005** | | **HIES 2010** | |
| --- | --- | --- | --- | --- | --- | --- |
|  | **Variable** | **Dataset** | **Variable** | **Dataset** | **Variable** | **Dataset** |
| Daily Consumption - 14 days |  |  |  |  |  |  |
| day 1 value |  |  |  |  | s09a1d_2 | rt015 |
| day 2 value |  |  |  |  | s09a1d_5 | rt015 |
| day 3 value |  |  |  |  | s09a1d_8 | rt015 |
| day 4 value |  |  |  |  | s09a1_11 | rt015 |
| day 5 value |  |  |  |  | s09a1_14 | rt015 |
| day 6 value |  |  |  |  | s09a1_17 | rt015 |
| day 7 value |  |  |  |  | s09a1_20 | rt015 |
| day 8 value |  |  |  |  | s09a1_23 | rt015 |
| day 9 value |  |  |  |  | s09a1_26 | rt015 |
| day 10 value |  |  |  |  | s09a1_29 | rt015 |
| day 11 value |  |  |  |  | s09a1_32 | rt015 |
| day 12 value |  |  |  |  | s09a1_35 | rt015 |
| day 13 value |  |  |  |  | s09a1_38 | rt015 |
| day 14 value |  |  |  |  | s09a1_41 | rt015 |
| food consumption 1-14 days | valu | S9A2 | valu | s9a2 |  |  |
| week 1 value |  |  |  |  | s09b1w_2 | rt016 |
| week 2 value |  |  |  |  | s09b1w_5 | rt016 |
| week 1 and week 2 value | valu | S9B | valu | s9b |  |  |
| monthly non-food expenditure | Q03_9C | S9C | q03_9c | s9c | s09c1_2 | rt017 |
| annual nonfood expenditure (clothes) | Q02_9D1 | S9D1 | q02_9d1 | s9d1 | s09d1_1 | rt018 |
| annual nonfood expenditure (everything else) | Q01_9D2 | S9D2 | q01_9d2 | s9d2 | s09d2_q0 | rt019 |
| OOP payments for healthcare | codes 381-392 and 401-413 | S9D2 | codes 391-402 and 411-423 | s9d2 | codes 451-463, 471-481 | rt019 |

Using HIES 2005 as an example:

**Total household expenditure** = total food expenditure + monthly nonfood expenditure + annual nonfood expenditure

- Total food expenditures = ((365.25/14*(total_9a + total_9b))
- Total monthly nonfood expenditure = 12*total_9c
- Total annual expenditure = total_9d1 +total_9d2 (includes annual OOP payments for healthcare)

**Total household nonfood expenditures** = monthly nonfood expenditure + annual nonfood expenditure

- Total monthly nonfood expenditure = 12*total_9c
- Total annual expenditure = total_9d1 +total_9d2 (includes annual OOP payments for healthcare)

Asset Index for HIES 2000 and 2010

The list of items used by the Demographic and Health Survey to create their wealth index was used to confirm the items to include in the asset index [2].

1. The Institue of Statistical Mathematics, Analysis SIIfCa. Users' manual for handling resampled micro data of Bangladesh Household Income and Expenditure Survey - Bangladesh HIES 2010 (Version 1.0) 2017 [Available from: <http://www.sinfonica.or.jp/information/research/bangladesh/Manual_Bangladesh_HIES_2010_v1.0.pdf>.

2. Demographic and Health Survey Program. Wealth Index Construction, Bangladesh 2011 2019 [Available from: <https://www.dhsprogram.com/programming/wealth%20index/Bangladesh%20DHS%202011/bangladesh%202011.pdf>.
