# Supplementary material for: Assessing the effects of disease-specific programs on health systems: An analysis of the Bangladesh Lymphatic Filariasis Elimination Program’s impacts on health service coverage and catastrophic health expenditure
Source: PLoS Negl Trop Dis. 2021 Nov 23;15(11):e0009894. doi: 10.1371/journal.pntd.0009894 (PMC8651132; doi:10.1371/journal.pntd.0009894)
Supplement: S3 File — (DOCX) [file pntd.0009894.s003.docx]

**Supplement 3. Estimating Equations**

Health service coverage rates

Equation 1 - *Y_it_ = β_0_* *+ β_1_T_i_ + β_2_DiD Estimator_it_* *+β_3_ Ended Treatment_it_ + αYear Fixed Effects_t_ +ε_it_*

Equation 2 - *Y_it_ = β_0_ + β_1_T_i_ + β_2_DiD Estimator_it_ + β_3_Ended Treatment_it_ +* *αYear Fixed Effects_t_ + β_4_5^th^ quintile_it_ + β_5_4^th^ quintile_it_ + β_6_2^nd^ quintile_it_ + β_7_1^st^ quintile_it_ + β_8_Rural_it_ + β_9_No Education_it_ + β_10_Primary_it_+ β_11_Secondary_it_+ β_12_15-19 years_it_ + β_13_20-24 years_it_ + β_14_25-29 years_it_ + β_15_30-34 years_it_ + β_16_35-39 years_it_ + β_17_40-44 years_it_ + β_18_Married_it_ + β_19_Unemployed_it_ + ε_it_*

Equation 3 - *Y_ijt_ = β_0_ + β_1_T_i_ + β_2_DiD Estimator_it_ +β_3_ Ended Treatment_it_+ αYear Fixed Effects_t_ + β_4_5^th^ quintile_it_ + … +* *β_19_Unemployed_it_ +* *γDistrict Fixed Effects_i_ + ε_ijt_*

*Rural vs Urban*

Equation 4 - *Y_it_ = β_0_ + β_1_T_i_ + β_2_DiD Estimator_it_ + β_3_ (DiD Estimator * Rural) _it_ +* *β_4_ Rural_it_ +* *β_5_Ended Treatment_it_ + αYear Fixed Effects_it_ +ε_it_*

Equation 5 - *Y_it_ = β_0_ + β_1_T_i_ + β_2_DiD Estimator_it_ + β_3_ (DiD Estimator * Rural) _it_ + β_4_ Rural_it_ + β_5_Ended Treatment_it_ + αYear Fixed Effects_it_ + β_6_5^th^ quintile_it_ + β_7_4^th^ quintile_it_ + β_8_2^nd^ quintile_it_ + β_9_1^st^ quintile_it_ + β_10_No Education_it_ + β_11_Primary_it_+ β_12_Secondary_it_+ β_13_15-19 years_it_ + β_14_20-24 years_it_ + β_15_25-29 years_it_ + β_16_30-34 years_it_ + β_17_35-39 years_it_ + β_18_40-44 years_it_ + β_19_Married_it_ + β_20_Unemployed_it_ + ε_it_*

Equation 6 - *Y_ijt_ = β_0_ + β_1_T_i_ + β_2_ DiD Estimator_it_ + β_3_ (DiD Estimator * Rural) _it_ + β_4_ Rural_it_ + β_5_Ended Treatment_it_ + αYear Fixed Effects_it_ +… + β_20_Unemployed_it_ + γDistrict Fixed Effects_i_+ ε_ijt_*

*Low vs high wealth quintiles*

Equation 7 - *Y_it_ = β_0_ + β_1_ T_i_ + β_2_ DiD Estimator_it_ + β_3_ (DiD Estimator * Low wealth quintiles) _it_ + β_4_ Low wealth quintiles_it_+ β_5_ Ended Treatment_it_ + αYear Fixed Effects_it_+ε_it_*

Equation 8 - *Y_it_ = β_0_ + β_1_ T_i_ + β_2_ DiD Estimator_it_ + β_3_ (DiD Estimator * Low wealth quintiles) _it_ + β_4_ Low wealth quintiles_it_ + β_5_ Ended Treatment_it_ + αYear Fixed Effects_it_ + β_6_Rural_it_ + β_7_No Education_it_ + β_8_Primary_it_+ β_9_Secondary_it_+ β_10_15-19 years_it_ + β_11_20-24 years_it_ + β_12_25-29 years_it_ + β_13_30-34 years_it_ + β_14_35-39 years_it_ + β_15_40-44 years_it_ + β_16_Married_it_ + β_17_Unemployed_it_ + ε_it_*

Equation 9 - *Y_ijt_ = β_0_ + β_1_T_i_ + β_2_ DiD Estimator_it_ + β_3_ (DiD Estimator * Low wealth quintiles) _it_ + β_4_ Low wealth quintiles_it_ + β_5_ Ended Treatment_it_ + αYear Fixed Effects_it_ +… + β_17_Unemployed_it_ + γDistrict Fixed Effects_i_ + ε_ijt_*

Catastrophic Health Expenditures

Equation 1 - *Y_it_ = β_0_ + β_1_T_i_ + β_2_DiD Estimator_it_ + αYear Fixed Effects_t_ + ε_it_*

Equation 2 - *Y_it_ = β_0_ + β_1_T_i_ + β_2_DiD Estimator_it_* *+ αYear Fixed Effects_t_ + β_3_Number of Household (HH) Members_it_ + β_4_1 Chronic Ill Mem._it_ + β_5_2 Chronic Ill Mem._it_ + β_6_3 or More Chronic Ill Mem._it_ + β_7_1^st^ quintile_it_ + β_8_2^nd^ quintile + β_9_4^th^ quintile_it_ + β_10_5^th^ quintile_it_ + β_11_Rural_it_ +* *β_12_Some Primary_it_+β_13_Primary_it_+ β_14_Some Secondary_it_+β_15_Secondary_it_ + β_16_Higher Education_it_ + β_17_Head HH Gender_it_ + β_18_Married_it_ + β_19_Employed_it_ + ε_it_*

Equation 3 - *Y_ijt_ = β_0_ + β_1_T_i_ + β_2_DiD Estimator_it_ + αYear Fixed Effects_t_ + β_3_Number of HH Members_it_ + … + β_19_Employed_it_ + γDistrict Fixed Effects_j_ + ε_ijt_*

*Rural vs Urban*

Equation 4 - *Y_it_ = β_0_ + β_1_T_i_ + β_2_DiD Estimator_it_ +* *β_3_ (DiD Estimator * Rural) _it_ + β_4_ Rural_it_ + αYear Fixed Effects_t_+ ε_it_*

Equation 5 - *Y_it_ = β_0_ + β_1_T_i_ + β_2_DiD Estimator_it_* *+ β_3_ (DiD Estimator * Rural) _it_ + β_4_ Rural_it_ + αYear Fixed Effects_t_+ β_5_Number of Household (HH) Members_it_ + β_6_1 Chronic Ill Mem._it_ + β_7_2 Chronic Ill Mem._it_ + β_8_3 or More Chronic Ill Mem._it_ + β_9_1^st^ quintile_it_ + β_10_2^nd^ quintile + β_11_4^th^ quintile_it_ + β_12_5^th^ quintile_it_ + β_13_Some Primary_it_+β_14_Primary_it_+ β_15_Some Secondary_it_+β_16_Secondary_it_ + β_17_Higher Education_it_ + β_18_Head HH Gender_it_ + β_19_Married_it_ + β_10_Employed_it_ + ε_it_*

Equation 6 - *Y_ijt_ = β_0_ + β_1_T_i_ + β_2_DiD Estimator_it_ + β_3_ (DiD Estimator * Rural) _it_ + β_4_ Rural_it_ + αYear Fixed Effects_t_ + β_5_Number of HH Members_it_ + … + β_20_Employed_it_ + γDistrict Fixed Effects_j_ + ε_ijt_*

*Low vs high wealth quintiles*

Equation 7 - *Y_it_ = β_0_ + β_1_T_i_ + β_2_DiD Estimator_it_ + β_3_ (DiD Estimator * Poor) _it_ + β_4_ Poor_it_ + αYear Fixed Effects_t_+ ε_it_*

Equation 8 - *Y_it_ = β_0_ + β_1_T_i_ + β_2_DiD Estimator_it_ + β_3_ (DiD Estimator * Poor) _it_ + β_4_ Poor_it_ + αYear Fixed Effects_t_ + β_5_Number of Household (HH) Members_it_ + β_6_1 Chronic Ill Mem._it_ + β_7_2 Chronic Ill Mem._it_ + β_8_3 or More Chronic Ill Mem._it_ + β_9_Some Primary_it_+β_10_Primary_it_+ β_11_Some Secondary_it_+β_12_Secondary_it_ + β_13_Higher Education_it_ + β_14_Head HH Gender_it_ + β_15_Married_it_ + β_16_Employed_it_ + β_17_ Rural_it_ + ε_it_*

Equation 9 - *Y_ijt_ = β_0_ + β_1_T_i_ + β_2_DiD Estimator_it_ + β_3_ (DiD Estimator * Poor) _it_ + β_4_ Poor _it_ + αYear Fixed Effects _t_ + β_5_Number of HH Members_it_ + … + β_17_Rural_it_ + γDistrict Fixed Effects_j_ + ε_ijt_*

*Text Box 1 – Terms of estimating equations*

- *Y_ijt_* represents the outcome of interest (e.g., CCI and CHE) for unit *i* of *j* in period *t*.

*i* represents the individual for outcome CCI and the household for outcome CHE.

*j* represents the district.

*t* represents the survey year.

- *β_0_* is the intercept.
- *β_1_* is the coefficient that captures the treatment effect.

*T_i_* = 1 if the individual lives in a treatment district.

- *β_2_* is the DiD estimator.
- *DiD* = the proportion of years that the MDA was implemented in a district from one survey year to the next.
- *β_3_* is the coefficient to differentiate between treatment districts that stopped treatment from one survey year to the next.

*Ended Treatment_it_* = 1 if the household is in a treatment district and treatment stops between survey years.

- *αYear Fixed Effects_t_* is perfectly collinear with the *Post* term in a traditional DiD estimating equation. It is equal to 1 after the MDA starts.
- *β_4-19_* are the coefficients for control variables four through 19 for outcome CCI.
- *β_3-19_* are the coefficients for control variables three through 19 for outcome CHE
- *γDistrict Fixed Effects_j_* is the coefficient to differentiate time-invariant characteristics of each district.
- ε*_ijt_* is the error term calculated with districts clustered.
