# Supplementary material for: Assessing the effects of disease-specific programs on health systems: An analysis of the Bangladesh Lymphatic Filariasis Elimination Program’s impacts on health service coverage and catastrophic health expenditure
Source: PLoS Negl Trop Dis. 2021 Nov 23;15(11):e0009894. doi: 10.1371/journal.pntd.0009894 (PMC8651132; doi:10.1371/journal.pntd.0009894)
Supplement: S4 File — (DOCX) [file pntd.0009894.s004.docx]

**Supplement 4. Results of Essential Health Service Coverage Rates DiD Analyses**

*Table 4-1. DiD analyses of Bangladesh's effect on CCI scores*

|  | Equation 1 – No Controls  OLS | Equation 2 – Controls  OLS | Equation 3 – Fixed Effects  OLS |
| --- | --- | --- | --- |
|  |  |  |  |
| Treatment district | 0.0140 (0.0153) | 0.0151 (0.00957) |  |
| DiD estimator | 0.00247 (0.0120) | 0.00837 (0.00964) | 0.00146 (0.00884) |
| MDA ended prior to subsequent survey year | 0.0201* (0.00904) | 0.0125+ (0.00739) | 0.00729 (0.00834) |
| Rural |  | -0.0397*** (0.00526) | -0.0385*** (0.00498) |
| Unemployed |  | -0.00274 (0.00505) | 0.00247 (0.00485) |
| Wealth quintile (Base: 3^rd^ quintile) |  |  |  |
| 5^th^ quintile (highest 20%) |  | 0.0732*** (0.00451) | 0.0734*** (0.00511) |
| 4^th^ quintile |  | 0.0224*** (0.00477) | 0.0230*** (0.00458) |
| 2^nd^ quintile |  | -0.0161*** (0.00379) | -0.0161*** (0.00378) |
| 1^st^ quintile (lowest 20%) |  | -0.0300*** (0.00456) | -0.0301*** (0.00429) |
| Education (Base: Higher education) |  |  |  |
| No education |  | -0.151*** (0.00986) | -0.147*** (0.00993) |
| Primary education completed |  | -0.115*** (0.00821) | -0.111*** (0.00847) |
| Secondary education completed |  | -0.0679*** (0.00651) | -0.0666*** (0.00657) |
| Married |  | 0.219*** (0.00902) | 0.215*** (0.00897) |
| Age (Base: 45-49 years) |  |  |  |
| 10-14 years |  | 0.00839 (0.0399) | -0.000220 (0.0403) |
| 15-19 years |  | 0.176*** (0.0156) | 0.168*** (0.0159) |
| 20-24 years |  | 0.173*** (0.0155) | 0.167*** (0.0157) |
| 25-29 years |  | 0.150*** (0.0148) | 0.144*** (0.0150) |
| 30-34 years |  | 0.132*** (0.0155) | 0.128*** (0.0158) |
| 35-39 years |  | 0.0969*** (0.0177) | 0.0941*** (0.0178) |
| 40-44 years |  | 0.0488** (0.0180) | 0.0481** (0.0180) |
| Observations | 32404 | 32404 | 32404 |
| Year Fixed Effects | Yes | Yes | Yes |
| District Fixed Effects | No | No | Yes |

+ p<.1, * p<.05, ** p<0.01, *** p<0.001 (Clustered standard errors in parentheses)

*Table 4-2.* *DiD analyses of the Bangladesh LFEP’s effect on whether* ***family planning*** *needs are met* *for women whose last-born child is five years or less*

|  | Equation 1 – No Controls | | Equation 2 – Controls | | Equation 3 – Fixed Effects | |
| --- | --- | --- | --- | --- | --- | --- |
|  | Probit | MFX | Probit | MFX | Probit | MFX |
|  |  |  |  |  |  |  |
| Treatment district | 0.159* (0.0626) | 0.0394* (0.0162) | 0.158** (0.0580) | 0.0388** (0.0146) |  |  |
| DiD estimator | 0.101 (0.0678) | 0.0251 (0.0169) | 0.105 (0.0666) | 0.0258 (0.0164) | 0.0433 (0.0684) | 0.0104 (0.0164) |
| MDA ended prior to subsequent survey year | 0.164** (0.0520) | 0.0408** (0.0130) | 0.145** (0.0490) | 0.0357** (0.0121) | 0.107* (0.0501) | 0.0257* (0.0120) |
| Rural |  |  | -0.220*** (0.0381) | -0.0540*** (0.00991) | -0.195*** (0.0322) | -0.0468*** (0.00769) |
| Unemployed |  |  | -0.211*** (0.0240) | -0.0519*** (0.00608) | -0.151*** (0.0231) | -0.0363*** (0.00552) |
| Wealth quintile (Base: 3^rd^ quintile) |  |  |  |  |  |  |
| 5^th^ quintile (highest 20%) |  |  | 0.0615 (0.0424) | 0.0151 (0.0104) | 0.0850+ (0.0436) | 0.0204+ (0.0105) |
| 4^th^ quintile |  |  | -0.0521+ (0.0315) | -0.0128+ (0.00773) | -0.0337 (0.0322) | -0.00808 (0.00772) |
| 2^nd^ quintile |  |  | 0.0142 (0.0278) | 0.00349 (0.00683) | -0.000714 (0.0278) | -0.000171 (0.00668) |
| 1^st^ quintile (lowest 20%) |  |  | -0.0270 (0.0305) | -0.00663 (0.00748) | -0.0525+ (0.0286) | -0.0126+ (0.00688) |
| Education (Base: Higher education) |  |  |  |  |  |  |
| No education |  |  | -0.175*** (0.0476) | -0.0430*** (0.0119) | -0.122** (0.0393) | -0.0293** (0.00943) |
| Primary education completed |  |  | -0.0649 (0.0412) | -0.0159 (0.0102) | -0.0231 (0.0396) | -0.00555 (0.00952) |
| Secondary education completed |  |  | -0.0653 (0.0438) | -0.0161 (0.0109) | -0.0399 (0.0416) | -0.00959 (0.00999) |
| Married^†^ |  |  | 0 (.) | 0 (.) | 0 (.) | 0 (.) |
| Age (Base: 45-49 years) |  |  |  |  |  |  |
| 10-14 years |  |  | -0.0208 (0.258) | -0.00510 (0.0633) | -0.101 (0.266) | -0.0242 (0.0639) |
| 15-19 years |  |  | 0.273** (0.0922) | 0.0670** (0.0224) | 0.215* (0.0931) | 0.0517* (0.0223) |
| 20-24 years |  |  | 0.311** (0.0994) | 0.0764** (0.0242) | 0.275** (0.102) | 0.0661** (0.0243) |
| 25-29 years |  |  | 0.300** (0.0986) | 0.0737** (0.0240) | 0.269** (0.102) | 0.0646** (0.0244) |
| 30-34 years |  |  | 0.292** (0.0969) | 0.0718** (0.0236) | 0.277** (0.101) | 0.0665** (0.0241) |
| 35-39 years |  |  | 0.212* (0.103) | 0.0521* (0.0252) | 0.206+ (0.107) | 0.0495+ (0.0257) |
| 40-44 years |  |  | 0.113 (0.101) | 0.0278 (0.0247) | 0.122 (0.102) | 0.0293 (0.0246) |
| Observations | 30608 | 30608 | 30458 | 30458 | 30458 | 30458 |
| Year Fixed Effects | Yes | Yes | Yes | Yes | Yes | Yes |
| District Fixed Effects | No | No | No | No | Yes | Yes |

+ p<.1, * p<.05, ** p<0.01, *** p<0.001 (Clustered standard errors in parentheses). †Standard errors were not estimable. Possibly because only married women were asked about family planning needs.

*Table 4-3. DiD analyses of the Bangladesh LFEP’s effect* *on whether women had at least* ***one ANC visit with a skilled provider*** *when pregnant with their last-born child, five years or less*

|  | Equation 1 – No Controls | | Equation 2 – Controls | | Equation 3 – Fixed Effects | |
| --- | --- | --- | --- | --- | --- | --- |
|  | Probit | MFX | Probit | MFX | Probit | MFX |
|  |  |  |  |  |  |  |
| Treatment district | 0.0798 (0.105) | 0.0301 (0.0398) | 0.136 (0.0843) | 0.0410 (0.0253) |  |  |
| DiD estimator | 0.0429 (0.0831) | 0.0162 (0.0314) | 0.119 (0.0862) | 0.0357 (0.0259) | 0.0159 (0.0609) | 0.00468 (0.0179) |
| MDA ended prior to subsequent survey year | 0.170* (0.0822) | 0.0642* (0.0311) | 0.0912 (0.0771) | 0.0274 (0.0232) | 0.0492 (0.0800) | 0.0144 (0.0235) |
| Rural |  |  | -0.361*** (0.0363) | -0.109*** (0.0105) | -0.346*** (0.0361) | -0.102*** (0.0105) |
| Unemployed |  |  | -0.0278 (0.0341) | -0.00837 (0.0103) | 0.0114 (0.0323) | 0.00334 (0.00948) |
| Wealth quintile (Base: 3^rd^ quintile) |  |  |  |  |  |  |
| 5^th^ quintile (highest 20%) |  |  | 0.673*** (0.0409) | 0.202*** (0.0122) | 0.644*** (0.0418) | 0.189*** (0.0119) |
| 4^th^ quintile |  |  | 0.249*** (0.0299) | 0.0748*** (0.00885) | 0.244*** (0.0301) | 0.0717*** (0.00878) |
| 2^nd^ quintile |  |  | -0.201*** (0.0268) | -0.0605*** (0.00783) | -0.203*** (0.0263) | -0.0596*** (0.00752) |
| 1^st^ quintile (lowest 20%) |  |  | -0.344*** (0.0384) | -0.104*** (0.0112) | -0.339*** (0.0345) | -0.0995*** (0.00988) |
| Education (Base: Higher education) |  |  |  |  |  |  |
| No education |  |  | -1.488*** (0.0723) | -0.447*** (0.0212) | -1.516*** (0.0693) | -0.445*** (0.0191) |
| Primary education completed |  |  | -1.227*** (0.0593) | -0.369*** (0.0177) | -1.230*** (0.0594) | -0.361*** (0.0169) |
| Secondary education completed |  |  | -0.792*** (0.0563) | -0.238*** (0.0176) | -0.804*** (0.0578) | -0.236*** (0.0172) |
| Married |  |  | 0.217** (0.0678) | 0.0653** (0.0205) | 0.208** (0.0647) | 0.0609** (0.0189) |
| Age (Base: 45-49 years) |  |  |  |  |  |  |
| 10-14 years |  |  | 0.587** (0.226) | 0.176** (0.0676) | 0.523* (0.232) | 0.154* (0.0679) |
| 15-19 years |  |  | 0.479*** (0.123) | 0.144*** (0.0373) | 0.415** (0.132) | 0.122** (0.0389) |
| 20-24 years |  |  | 0.435*** (0.118) | 0.131*** (0.0357) | 0.390** (0.126) | 0.115** (0.0372) |
| 25-29 years |  |  | 0.413*** (0.117) | 0.124*** (0.0355) | 0.373** (0.127) | 0.109** (0.0375) |
| 30-34 years |  |  | 0.365** (0.125) | 0.110** (0.0378) | 0.339* (0.134) | 0.0996* (0.0395) |
| 35-39 years |  |  | 0.304* (0.130) | 0.0914* (0.0392) | 0.285* (0.138) | 0.0836* (0.0405) |
| 40-44 years |  |  | 0.151 (0.149) | 0.0454 (0.0448) | 0.142 (0.159) | 0.0417 (0.0467) |
| Observations | 24454 | 24454 | 24454 | 24454 | 24454 | 24454 |
| Year Fixed Effects | Yes | Yes | Yes | Yes | Yes | Yes |
| District Fixed Effects | No | No | No | No | Yes | Yes |

+ p<.1, * p<.05, ** p<0.01, *** p<0.001 (Clustered standard errors in parentheses)

*Table 4-4. DiD analyses of the Bangladesh LFEP’s effect on whether women’s* ***delivery was assisted by an SBA***

|  | Equation 1 – No Controls | | Equation 2 – Controls | | Equation 3 – Fixed Effects | |
| --- | --- | --- | --- | --- | --- | --- |
|  | Probit | MFX | Probit | MFX | Probit | MFX |
|  |  |  |  |  |  |  |
| Treatment district | 0.0268 (0.0935) | 0.00894 (0.0310) | 0.103+ (0.0604) | 0.0274+ (0.0161) |  |  |
| DiD estimator | 0.00338 (0.0671) | 0.00113 (0.0223) | 0.0324 (0.0673) | 0.00858 (0.0178) | 0.0959+ (0.0525) | 0.0250+ (0.0137) |
| MDA ended prior to subsequent survey year | 0.135 (0.0914) | 0.0448 (0.0305) | 0.0736 (0.0595) | 0.0195 (0.0158) | 0.0576 (0.0593) | 0.0150 (0.0155) |
| Rural |  |  | -0.363*** (0.0366) | -0.0962*** (0.00952) | -0.366*** (0.0412) | -0.0955*** (0.0108) |
| Unemployed |  |  | 0.131** (0.0427) | 0.0348** (0.0113) | 0.155*** (0.0418) | 0.0403*** (0.0108) |
| Wealth quintile (Base: 3^rd^ quintile) |  |  |  |  |  |  |
| 5^th^ quintile (highest 20%) |  |  | 0.629*** (0.0404) | 0.167*** (0.0101) | 0.599*** (0.0423) | 0.156*** (0.0106) |
| 4^th^ quintile |  |  | 0.206*** (0.0275) | 0.0545*** (0.00722) | 0.195*** (0.0280) | 0.0508*** (0.00728) |
| 2^nd^ quintile |  |  | -0.193*** (0.0361) | -0.0512*** (0.00968) | -0.181*** (0.0360) | -0.0470*** (0.00938) |
| 1^st^ quintile (lowest 20%) |  |  | -0.264*** (0.0412) | -0.0699*** (0.0109) | -0.250*** (0.0383) | -0.0652*** (0.00999) |
| Education (Base: Higher education) |  |  |  |  |  |  |
| No education |  |  | -1.355*** (0.0835) | -0.359*** (0.0206) | -1.373*** (0.0827) | -0.358*** (0.0197) |
| Primary education completed |  |  | -1.179*** (0.0694) | -0.312*** (0.0171) | -1.192*** (0.0683) | -0.311*** (0.0162) |
| Secondary education completed |  |  | -0.765*** (0.0465) | -0.203*** (0.0119) | -0.783*** (0.0462) | -0.204*** (0.0113) |
| Married |  |  | -0.0130 (0.0630) | -0.00344 (0.0167) | -0.0210 (0.0672) | -0.00546 (0.0175) |
| Age (Base: 45-49 years) |  |  |  |  |  |  |
| 10-14 years |  |  | 0.517+ (0.275) | 0.137+ (0.0732) | 0.391 (0.270) | 0.102 (0.0705) |
| 15-19 years |  |  | 0.383** (0.146) | 0.102** (0.0387) | 0.332* (0.147) | 0.0866* (0.0382) |
| 20-24 years |  |  | 0.337* (0.146) | 0.0894* (0.0387) | 0.293* (0.147) | 0.0764* (0.0381) |
| 25-29 years |  |  | 0.357* (0.147) | 0.0946* (0.0389) | 0.319* (0.147) | 0.0830* (0.0383) |
| 30-34 years |  |  | 0.345* (0.146) | 0.0915* (0.0386) | 0.316* (0.147) | 0.0825* (0.0381) |
| 35-39 years |  |  | 0.372* (0.145) | 0.0986** (0.0383) | 0.347* (0.146) | 0.0906* (0.0377) |
| 40-44 years |  |  | 0.203 (0.158) | 0.0538 (0.0417) | 0.183 (0.159) | 0.0478 (0.0414) |
| Observations | 26070 | 26070 | 26070 | 26070 | 26070 | 26070 |
| Year Fixed Effects | Yes | Yes | Yes | Yes | Yes | Yes |
| District Fixed Effects | No | No | No | No | Yes | Yes |

+ p<.1, * p<.05, ** p<0.01, *** p<0.001 (Clustered standard errors in parentheses)

*Table 4-5. DiD analyses of the Bangladesh LFEP’s effect on children, five years or less, receiving a* ***BCG vaccine***

|  | Equation 1 – No Controls | | Equation 2 – Controls | | Equation 3 – Fixed Effects | |
| --- | --- | --- | --- | --- | --- | --- |
|  | Probit | MFX | Probit | MFX | Probit | MFX |
|  |  |  |  |  |  |  |
| Treatment district | 0.154* (0.0742) | 0.0184* (0.00922) | 0.153* (0.0637) | 0.0177* (0.00760) |  |  |
| DiD estimator | 0.0713 (0.0884) | 0.00852 (0.0106) | 0.0956 (0.0822) | 0.0111 (0.00958) | 0.0516 (0.0873) | 0.00586 (0.00991) |
| MDA ended prior to subsequent survey year | 0.00348 (0.0702) | 0.000417 (0.00840) | -0.0247 (0.0631) | -0.00286 (0.00726) | -0.0560 (0.0630) | -0.00636 (0.00715) |
| Rural |  |  | -0.0205 (0.0340) | -0.00238 (0.00397) | -0.00351 (0.0299) | -0.000398 (0.00339) |
| Unemployed |  |  | -0.157*** (0.0372) | -0.0181*** (0.00444) | -0.129*** (0.0354) | -0.0147*** (0.00401) |
| Wealth quintile (Base: 3^rd^ quintile) |  |  |  |  |  |  |
| 5^th^ quintile (highest 20%) |  |  | 0.159** (0.0590) | 0.0184** (0.00698) | 0.191*** (0.0553) | 0.0217*** (0.00628) |
| 4^th^ quintile |  |  | 0.0485 (0.0439) | 0.00560 (0.00515) | 0.0539 (0.0434) | 0.00612 (0.00492) |
| 2^nd^ quintile |  |  | -0.0155 (0.0401) | -0.00179 (0.00462) | -0.0233 (0.0410) | -0.00264 (0.00465) |
| 1^st^ quintile (lowest 20%) |  |  | -0.122** (0.0423) | -0.0141** (0.00503) | -0.127** (0.0473) | -0.0144** (0.00537) |
| Education (Base: Higher education) |  |  |  |  |  |  |
| No education |  |  | -0.326*** (0.0542) | -0.0377*** (0.00670) | -0.260*** (0.0586) | -0.0295*** (0.00665) |
| Primary education completed |  |  | -0.0591 (0.0582) | -0.00683 (0.00678) | -0.00682 (0.0586) | -0.000774 (0.00666) |
| Secondary education completed |  |  | 0.0574 (0.0530) | 0.00663 (0.00614) | 0.0866 (0.0554) | 0.00984 (0.00628) |
| Married |  |  | 0.171* (0.0744) | 0.0198* (0.00876) | 0.141+ (0.0751) | 0.0160+ (0.00852) |
| Age (Base: 45-49 years) |  |  |  |  |  |  |
| 10-14 years |  |  | -0.992*** (0.257) | -0.115*** (0.0284) | -1.061*** (0.259) | -0.120*** (0.0294) |
| 15-19 years |  |  | -0.159 (0.115) | -0.0183 (0.0131) | -0.221* (0.113) | -0.0251* (0.0128) |
| 20-24 years |  |  | 0.175+ (0.104) | 0.0202 (0.0123) | 0.136 (0.101) | 0.0154 (0.0115) |
| 25-29 years |  |  | 0.190+ (0.110) | 0.0220+ (0.0130) | 0.158 (0.107) | 0.0179 (0.0122) |
| 30-34 years |  |  | 0.144 (0.0992) | 0.0166 (0.0117) | 0.121 (0.0973) | 0.0138 (0.0110) |
| 35-39 years |  |  | 0.0480 (0.1000) | 0.00555 (0.0116) | 0.0332 (0.0975) | 0.00377 (0.0111) |
| 40-44 years |  |  | -0.0405 (0.134) | -0.00468 (0.0155) | -0.0516 (0.132) | -0.00586 (0.0150) |
| Observations | 28888 | 28888 | 28888 | 28888 | 28888 | 28888 |
| Year Fixed Effects | Yes | Yes | Yes | Yes | Yes | Yes |
| District Fixed Effects | No | No | No | No | Yes | Yes |

+ p<.1, * p<.05, ** p<0.01, *** p<0.001 (Clustered standard errors in parentheses)

*Table 4-6. DiD analyses of the Bangladesh LFEP’s effect on children, five years or less, receiving* ***three doses of DPT vaccine***

|  | Equation 1 – No Controls | | Equation 2 – Controls | | Equation 3 – Fixed Effects | |
| --- | --- | --- | --- | --- | --- | --- |
|  | Probit | MFX | Probit | MFX | Probit | MFX |
|  |  |  |  |  |  |  |
| Treatment district | 0.0549 (0.0653) | 0.0138 (0.0166) | 0.0581 (0.0554) | 0.0142 (0.0136) |  |  |
| DiD estimator | 0.0327 (0.0748) | 0.00825 (0.0189) | 0.0589 (0.0707) | 0.0143 (0.0172) | 0.0202 (0.0731) | 0.00484 (0.0176) |
| MDA ended prior to subsequent survey year | 0.0800 (0.0576) | 0.0202 (0.0146) | 0.0582 (0.0519) | 0.0142 (0.0127) | 0.0424 (0.0554) | 0.0102 (0.0133) |
| Rural |  |  | -0.00682 (0.0269) | -0.00166 (0.00656) | -0.00256 (0.0269) | -0.000615 (0.00646) |
| Unemployed |  |  | -0.122*** (0.0327) | -0.0298*** (0.00805) | -0.0996*** (0.0297) | -0.0239*** (0.00716) |
| Wealth quintile (Base: 3^rd^ quintile) |  |  |  |  |  |  |
| 5^th^ quintile (highest 20%) |  |  | 0.147*** (0.0329) | 0.0358*** (0.00830) | 0.174*** (0.0361) | 0.0418*** (0.00865) |
| 4^th^ quintile |  |  | 0.0217 (0.0364) | 0.00529 (0.00887) | 0.0297 (0.0364) | 0.00714 (0.00873) |
| 2^nd^ quintile |  |  | -0.00789 (0.0294) | -0.00192 (0.00716) | -0.00457 (0.0299) | -0.00110 (0.00718) |
| 1^st^ quintile (lowest 20%) |  |  | -0.110*** (0.0274) | -0.0268*** (0.00681) | -0.0998*** (0.0303) | -0.0240*** (0.00725) |
| Education (Base: Higher education) |  |  |  |  |  |  |
| No education |  |  | -0.346*** (0.0587) | -0.0842*** (0.0145) | -0.288*** (0.0602) | -0.0692*** (0.0144) |
| Primary education completed |  |  | -0.101+ (0.0571) | -0.0247+ (0.0140) | -0.0540 (0.0585) | -0.0130 (0.0140) |
| Secondary education completed |  |  | 0.0461 (0.0454) | 0.0112 (0.0111) | 0.0657 (0.0493) | 0.0158 (0.0118) |
| Married |  |  | 0.0850 (0.0681) | 0.0207 (0.0166) | 0.0650 (0.0680) | 0.0156 (0.0163) |
| Age (Base: 45-49 years) |  |  |  |  |  |  |
| 10-14 years |  |  | -1.553*** (0.261) | -0.378*** (0.0633) | -1.620*** (0.264) | -0.389*** (0.0632) |
| 15-19 years |  |  | -0.352** (0.108) | -0.0858** (0.0263) | -0.397*** (0.110) | -0.0954*** (0.0265) |
| 20-24 years |  |  | 0.0560 (0.101) | 0.0136 (0.0246) | 0.0293 (0.103) | 0.00705 (0.0247) |
| 25-29 years |  |  | 0.0989 (0.106) | 0.0241 (0.0258) | 0.0770 (0.107) | 0.0185 (0.0258) |
| 30-34 years |  |  | 0.0989 (0.105) | 0.0241 (0.0256) | 0.0844 (0.106) | 0.0203 (0.0255) |
| 35-39 years |  |  | 0.0600 (0.116) | 0.0146 (0.0283) | 0.0537 (0.116) | 0.0129 (0.0280) |
| 40-44 years |  |  | 0.0319 (0.124) | 0.00777 (0.0302) | 0.0339 (0.124) | 0.00815 (0.0299) |
| Observations | 28892 | 28892 | 28892 | 28892 | 28892 | 28892 |
| Year Fixed Effects | Yes | Yes | Yes | Yes | Yes | Yes |
| District Fixed Effects | No | No | No | No | Yes | Yes |

+ p<.1, * p<.05, ** p<0.01, *** p<0.001 (Clustered standard errors in parentheses)

*Table 4-7. DiD analyses of the Bangladesh LFEP’s effect on children, five years or less, receiving a* ***measles vaccine***

|  | Equation 1 – No Controls | | Equation 2 – Controls | | Equation 3 – Fixed Effects | |
| --- | --- | --- | --- | --- | --- | --- |
|  | Probit | MFX | Probit | MFX | Probit | MFX |
|  |  |  |  |  |  |  |
| Treatment district | 0.0405 (0.0512) | 0.0128 (0.0162) | 0.0497 (0.0461) | 0.0152 (0.0141) |  |  |
| DiD estimator | 0.0172 (0.0557) | 0.00545 (0.0177) | 0.0416 (0.0542) | 0.0127 (0.0166) | 0.0223 (0.0547) | 0.00675 (0.0166) |
| MDA ended prior to subsequent survey year | 0.0395 (0.0490) | 0.0125 (0.0155) | 0.0213 (0.0445) | 0.00649 (0.0136) | 0.00178 (0.0434) | 0.000540 (0.0132) |
| Rural |  |  | 0.0253 (0.0269) | 0.00771 (0.00822) | 0.0116 (0.0232) | 0.00350 (0.00702) |
| Unemployed |  |  | -0.107*** (0.0297) | -0.0327*** (0.00909) | -0.0987*** (0.0281) | -0.0299*** (0.00852) |
| Wealth quintile (Base: 3^rd^ quintile) |  |  |  |  |  |  |
| 5^th^ quintile (highest 20%) |  |  | 0.0880*** (0.0267) | 0.0269** (0.00826) | 0.120*** (0.0297) | 0.0364*** (0.00901) |
| 4^th^ quintile |  |  | 0.0117 (0.0287) | 0.00359 (0.00877) | 0.0201 (0.0287) | 0.00610 (0.00870) |
| 2^nd^ quintile |  |  | -0.0123 (0.0284) | -0.00376 (0.00868) | -0.0106 (0.0275) | -0.00322 (0.00835) |
| 1^st^ quintile (lowest 20%) |  |  | -0.111*** (0.0268) | -0.0340*** (0.00819) | -0.112*** (0.0269) | -0.0340*** (0.00811) |
| Education (Base: Higher education) |  |  |  |  |  |  |
| No education |  |  | -0.260*** (0.0448) | -0.0794*** (0.0140) | -0.218*** (0.0449) | -0.0660*** (0.0135) |
| Primary education completed |  |  | -0.0605 (0.0421) | -0.0185 (0.0129) | -0.0204 (0.0415) | -0.00618 (0.0126) |
| Secondary education completed |  |  | 0.0590+ (0.0358) | 0.0180+ (0.0109) | 0.0756* (0.0370) | 0.0229* (0.0112) |
| Married |  |  | 0.0428 (0.0579) | 0.0131 (0.0177) | 0.0307 (0.0579) | 0.00930 (0.0175) |
| Age (Base: 45-49 years) |  |  |  |  |  |  |
| 10-14 years |  |  | -2.106*** (0.329) | -0.643*** (0.0998) | -2.150*** (0.330) | -0.651*** (0.0996) |
| 15-19 years |  |  | -0.602*** (0.119) | -0.184*** (0.0362) | -0.637*** (0.119) | -0.193*** (0.0361) |
| 20-24 years |  |  | -0.0948 (0.114) | -0.0289 (0.0348) | -0.116 (0.114) | -0.0353 (0.0345) |
| 25-29 years |  |  | -0.0199 (0.121) | -0.00609 (0.0368) | -0.0380 (0.120) | -0.0115 (0.0363) |
| 30-34 years |  |  | 0.0187 (0.115) | 0.00570 (0.0351) | 0.00469 (0.114) | 0.00142 (0.0346) |
| 35-39 years |  |  | 0.00235 (0.125) | 0.000718 (0.0383) | -0.00344 (0.125) | -0.00104 (0.0378) |
| 40-44 years |  |  | -0.0339 (0.115) | -0.0104 (0.0352) | -0.0414 (0.116) | -0.0125 (0.0350) |
| Observations | 28867 | 28867 | 28867 | 28867 | 28867 | 28867 |
| Year Fixed Effects | Yes | Yes | Yes | Yes | Yes | Yes |
| District Fixed Effects | No | No | No | No | Yes | Yes |

+ p<.1, * p<.05, ** p<0.01, *** p<0.001 (Clustered standard errors in parentheses)

*Table 4-8.* *DiD analyses of the Bangladesh LFEP’s effect on care-seeking behaviors of mothers who sought treatment for their last-born child, five years or less, with an* ***ARI***

|  | Equation 1 – No Controls | | Equation 2 – Controls | | Equation 3 – Fixed Effects | |
| --- | --- | --- | --- | --- | --- | --- |
|  | Probit | MFX | Probit | MFX | Probit | MFX |
|  |  |  |  |  |  |  |
| Treatment district | -0.0361 (0.0772) | -0.0133 (0.0283) | -0.0125 (0.0523) | -0.00426 (0.0178) |  |  |
| DiD estimator | -0.0596 (0.0787) | -0.0219 (0.0289) | -0.0127 (0.0773) | -0.00431 (0.0263) | -0.0206 (0.0800) | -0.00688 (0.0267) |
| MDA ended prior to subsequent survey year | 0.121 (0.0737) | 0.0443 (0.0271) | 0.0766 (0.0824) | 0.0260 (0.0280) | 0.102 (0.0920) | 0.0339 (0.0307) |
| Rural |  |  | -0.240*** (0.0451) | -0.0816*** (0.0152) | -0.234*** (0.0490) | -0.0781*** (0.0162) |
| Unemployed |  |  | 0.0602 (0.0513) | 0.0205 (0.0174) | 0.0554 (0.0516) | 0.0185 (0.0172) |
| Wealth quintile (Base: 3^rd^ quintile) |  |  |  |  |  |  |
| 5^th^ quintile (highest 20%) |  |  | 0.478*** (0.0522) | 0.163*** (0.0175) | 0.465*** (0.0525) | 0.155*** (0.0174) |
| 4^th^ quintile |  |  | 0.154*** (0.0453) | 0.0523*** (0.0155) | 0.150** (0.0459) | 0.0501** (0.0154) |
| 2^nd^ quintile |  |  | -0.155*** (0.0464) | -0.0527*** (0.0158) | -0.147** (0.0478) | -0.0493** (0.0160) |
| 1^st^ quintile (lowest 20%) |  |  | -0.242*** (0.0570) | -0.0823*** (0.0192) | -0.233*** (0.0592) | -0.0779*** (0.0195) |
| Education (Base: Higher education) |  |  |  |  |  |  |
| No education |  |  | -0.441*** (0.0919) | -0.150*** (0.0312) | -0.446*** (0.0893) | -0.149*** (0.0298) |
| Primary education completed |  |  | -0.327*** (0.0842) | -0.111*** (0.0286) | -0.340*** (0.0821) | -0.114*** (0.0274) |
| Secondary education completed |  |  | -0.200** (0.0742) | -0.0679** (0.0252) | -0.205** (0.0751) | -0.0686** (0.0251) |
| Married |  |  | -0.104 (0.0894) | -0.0355 (0.0304) | -0.0909 (0.0891) | -0.0304 (0.0298) |
| Age (Base: 45-49 years) |  |  |  |  |  |  |
| 10-14 years |  |  | -0.159 (0.416) | -0.0542 (0.141) | -0.204 (0.428) | -0.0682 (0.143) |
| 15-19 years |  |  | 0.208 (0.265) | 0.0708 (0.0900) | 0.229 (0.276) | 0.0764 (0.0921) |
| 20-24 years |  |  | 0.122 (0.266) | 0.0414 (0.0905) | 0.144 (0.278) | 0.0482 (0.0929) |
| 25-29 years |  |  | 0.160 (0.263) | 0.0545 (0.0894) | 0.177 (0.275) | 0.0593 (0.0918) |
| 30-34 years |  |  | 0.0949 (0.280) | 0.0323 (0.0952) | 0.0988 (0.290) | 0.0330 (0.0970) |
| 35-39 years |  |  | 0.0741 (0.256) | 0.0252 (0.0870) | 0.0898 (0.270) | 0.0300 (0.0901) |
| 40-44 years |  |  | -0.113 (0.262) | -0.0384 (0.0890) | -0.0861 (0.273) | -0.0288 (0.0912) |
| Observations | 7887 | 7887 | 7887 | 7887 | 7887 | 7887 |
| Year Fixed Effects | Yes | Yes | Yes | Yes | Yes | Yes |
| District Fixed Effects | No | No | No | No | Yes | Yes |

+ p<.1, * p<.05, ** p<0.01, *** p<0.001 (Clustered standard errors in parentheses)

*Table 4-9. DiD analyses of the Bangladesh LFEP’s effect on care-seeking behaviors of mothers who sought treatment for their last-born child, five years or less, with* ***diarrhea***

|  | Equation 1 – No Controls | | Equation 2 – Controls | | Equation 3 – Fixed Effects | |
| --- | --- | --- | --- | --- | --- | --- |
|  | Probit | MFX | Probit | MFX | Probit | MFX |
|  |  |  |  |  |  |  |
| Treatment district | -0.311** (0.117) | -0.109** (0.0408) | -0.329** (0.120) | -0.113** (0.0414) |  |  |
| DiD estimator | 0.178 (0.149) | 0.0623 (0.0523) | 0.198 (0.147) | 0.0683 (0.0507) | 0.104 (0.152) | 0.0350 (0.0511) |
| MDA ended prior to subsequent survey year | 0.142 (0.165) | 0.0498 (0.0579) | 0.123 (0.165) | 0.0424 (0.0568) | 0.0104 (0.193) | 0.00350 (0.0649) |
| Rural |  |  | 0.0875 (0.0682) | 0.0301 (0.0234) | 0.0968 (0.0757) | 0.0325 (0.0254) |
| Unemployed |  |  | -0.0131 (0.0780) | -0.00451 (0.0269) | -0.00680 (0.0824) | -0.00228 (0.0277) |
| Wealth quintile (Base: 3^rd^ quintile) |  |  |  |  |  |  |
| 5^th^ quintile (highest 20%) |  |  | 0.0207 (0.0936) | 0.00715 (0.0323) | 0.0491 (0.103) | 0.0165 (0.0347) |
| 4^th^ quintile |  |  | -0.0496 (0.101) | -0.0171 (0.0348) | -0.0289 (0.102) | -0.00971 (0.0342) |
| 2^nd^ quintile |  |  | -0.138 (0.0847) | -0.0477 (0.0290) | -0.150+ (0.0861) | -0.0505+ (0.0287) |
| 1^st^ quintile (lowest 20%) |  |  | -0.241** (0.0891) | -0.0831** (0.0306) | -0.274** (0.0922) | -0.0920** (0.0306) |
| Education (Base: Higher education) |  |  |  |  |  |  |
| No education |  |  | -0.323* (0.141) | -0.111* (0.0486) | -0.357* (0.148) | -0.120* (0.0499) |
| Primary education completed |  |  | -0.288* (0.128) | -0.0993* (0.0444) | -0.312* (0.133) | -0.105* (0.0449) |
| Secondary education completed |  |  | -0.166 (0.159) | -0.0572 (0.0547) | -0.200 (0.161) | -0.0673 (0.0545) |
| Married |  |  | 0.0674 (0.210) | 0.0232 (0.0725) | 0.0372 (0.215) | 0.0125 (0.0722) |
| Age (Base: 45-49 years) |  |  |  |  |  |  |
| 10-14 years |  |  | 0.602 (0.755) | 0.207 (0.260) | 0.430 (0.763) | 0.144 (0.256) |
| 15-19 years |  |  | 0.294 (0.404) | 0.101 (0.139) | 0.191 (0.416) | 0.0640 (0.140) |
| 20-24 years |  |  | 0.196 (0.403) | 0.0674 (0.139) | 0.107 (0.414) | 0.0360 (0.139) |
| 25-29 years |  |  | 0.207 (0.406) | 0.0712 (0.140) | 0.107 (0.416) | 0.0361 (0.140) |
| 30-34 years |  |  | 0.142 (0.411) | 0.0490 (0.142) | 0.0302 (0.426) | 0.0102 (0.143) |
| 35-39 years |  |  | -0.111 (0.442) | -0.0383 (0.152) | -0.213 (0.454) | -0.0716 (0.152) |
| 40-44 years |  |  | 0.578 (0.471) | 0.199 (0.162) | 0.492 (0.477) | 0.165 (0.161) |
| Observations | 2114 | 2114 | 2114 | 2114 | 2112 | 2112 |
| Year Fixed Effects | Yes | Yes | Yes | Yes | Yes | Yes |
| District Fixed Effects | No | No | No | No | Yes | Yes |

+ p<.1, * p<.05, ** p<0.01, *** p<0.001 (Clustered standard errors in parentheses)
