# Supplementary material for: Assessing the effects of disease-specific programs on health systems: An analysis of the Bangladesh Lymphatic Filariasis Elimination Program’s impacts on health service coverage and catastrophic health expenditure
Source: PLoS Negl Trop Dis. 2021 Nov 23;15(11):e0009894. doi: 10.1371/journal.pntd.0009894 (PMC8651132; doi:10.1371/journal.pntd.0009894)
Supplement: S5 File — (DOCX) [file pntd.0009894.s005.docx]

**Supplement 5. Results of CHE incidence DiD Analyses**

*Table 5-1. DiD analyses of the Bangladesh LFEP’s effect on incidence of CHE in treatment districts, using* ***5% of THE*** *as a threshold*

|  | Equation 1 – No Controls | | Equation 2 – With Controls | | Equation 3 – Fixed Effects | |
| --- | --- | --- | --- | --- | --- | --- |
|  | Logit | MFX | Logit | MFX | Logit | MFX |
|  |  |  |  |  |  |  |
| Treatment district | 0.0214 (0.165) | 0.00413 (0.0318) | 0.00734 (0.171) | 0.00138 (0.0322) |  |  |
| DiD estimator | -0.439+ (0.249) | -0.0849+ (0.0481) | -0.458+ (0.256) | -0.0863+ (0.0483) | -0.379 (0.246) | -0.0687 (0.0446) |
| Number of chronically ill household members (Reference: 0 household members) |  |  |  |  |  |  |
| 1 household member |  |  | 0.466*** (0.0595) | 0.0878*** (0.0112) | 0.470*** (0.0652) | 0.0853*** (0.0116) |
| 2 household members |  |  | 0.713*** (0.0741) | 0.134*** (0.0140) | 0.717*** (0.0806) | 0.130*** (0.0141) |
| More than 2 household members |  |  | 0.998*** (0.0965) | 0.188*** (0.0175) | 0.981*** (0.0901) | 0.178*** (0.0156) |
| Number of household members |  |  | -0.0520*** (0.0134) | -0.00980*** (0.00258) | -0.061*** (0.0111) | -0.0111*** (0.00199) |
| Rural |  |  | 0.124+ (0.0749) | 0.0235+ (0.0139) | 0.0813 (0.0520) | 0.0148 (0.00941) |
| HH working for a livelihood |  |  | -0.352*** (0.0563) | -0.0663*** (0.0111) | -0.342*** (0.0522) | -0.0622*** (0.00949) |
| Female HH |  |  | -0.154** (0.0546) | -0.0290** (0.0104) | -0.152** (0.0518) | -0.0276** (0.00944) |
| Wealth quintile (Base: 3^rd^ quintile) |  |  |  |  |  |  |
| 5^th^ quintile (highest 20%) |  |  | -0.145** (0.0519) | -0.0274** (0.00964) | -0.132** (0.0440) | -0.0239** (0.00796) |
| 4^th^ quintile |  |  | -0.0548 (0.0527) | -0.0103 (0.00985) | -0.0510 (0.0507) | -0.00926 (0.00918) |
| 2^nd^ quintile |  |  | 0.0147 (0.0455) | 0.00277 (0.00858) | 0.00265 (0.0477) | 0.000481 (0.00867) |
| 1^st^ quintile (lowest 20%) |  |  | 0.115* (0.0539) | 0.0216* (0.0101) | 0.0826+ (0.0496) | 0.0150+ (0.00901) |
| Married HH |  |  | 0.0288 (0.0691) | 0.00543 (0.0130) | 0.0528 (0.0676) | 0.00959 (0.0123) |
| HH education level (Reference: No formal education) |  |  |  |  |  |  |
| Some primary education |  |  | -0.178** (0.0602) | -0.0335** (0.0114) | -0.182** (0.0645) | -0.0330** (0.0117) |
| Primary education |  |  | -0.114* (0.0513) | -0.0216* (0.00988) | -0.138** (0.0477) | -0.0251** (0.00870) |
| Some secondary education |  |  | 0.00364 (0.0503) | 0.000686 (0.00948) | -0.0208 (0.0437) | -0.00377 (0.00794) |
| Secondary education |  |  | -0.0177 (0.0721) | -0.00333 (0.0136) | -0.0617 (0.0645) | -0.0112 (0.0117) |
| Higher education |  |  | -0.0243 (0.0684) | -0.00458 (0.0129) | -0.0431 (0.0657) | -0.00783 (0.0119) |
| Observations | 29420 | 29420 | 29303 | 29303 | 29303 | 29303 |
| Year Fixed Effects | Yes | Yes | Yes | Yes | Yes | Yes |
| District Fixed Effects | No | No | No | No | Yes | Yes |

+ p<.1, * p<.05, ** p<0.01, *** p<0.001 (Clustered standard errors in parentheses).

*Table 5-2.* *DiD analyses of the Bangladesh LFEP’s effect on incidence of CHE in treatment districts using* ***10% of THE*** *as a threshold*

|  | Equation 1 – No Controls | | Equation 2 – With Controls | | Equation 3 – Fixed Effects | |
| --- | --- | --- | --- | --- | --- | --- |
|  | Logit | MFX | Logit | MFX | Logit | MFX |
|  |  |  |  |  |  |  |
| Treatment district | 0.0647 (0.230) | 0.00793 (0.0282) | 0.0606 (0.237) | 0.00737 (0.0288) |  |  |
| DiD estimator | -0.962** (0.322) | -0.118** (0.0419) | -0.969** (0.329) | -0.118** (0.0425) | -0.828** (0.293) | -0.0966** (0.0343) |
| Number of chronically ill household members (Reference: 0 household members) |  |  |  |  |  |  |
| 1 household member |  |  | 0.0894 (0.0895) | 0.0109 (0.0107) | 0.0902 (0.0956) | 0.0105 (0.0112) |
| 2 household members |  |  | 0.150 (0.110) | 0.0183 (0.0131) | 0.173 (0.118) | 0.0202 (0.0138) |
| More than 2 household members |  |  | 0.437*** (0.118) | 0.0531*** (0.0139) | 0.464*** (0.114) | 0.0541*** (0.0133) |
| Number of household members |  |  | -0.0674*** (0.0163) | -0.00819*** (0.00197) | -0.0821*** (0.0114) | -0.00958*** (0.00133) |
| Rural |  |  | -0.0371 (0.0840) | -0.00450 (0.0103) | -0.0676 (0.0647) | -0.00789 (0.00755) |
| HH working for a livelihood |  |  | -0.389*** (0.0621) | -0.0473*** (0.00793) | -0.363*** (0.0602) | -0.0423*** (0.00713) |
| Female HH |  |  | -0.200* (0.0791) | -0.0243* (0.00946) | -0.217** (0.0714) | -0.0253** (0.00835) |
| Wealth quintile (Base: 3^rd^ quintile) |  |  |  |  |  |  |
| 5^th^ quintile (highest 20%) |  |  | -0.0850 (0.0647) | -0.0103 (0.00778) | -0.0574 (0.0624) | -0.00670 (0.00727) |
| 4^th^ quintile |  |  | -0.0179 (0.0721) | -0.00217 (0.00873) | -0.00566 (0.0696) | -0.000661 (0.00812) |
| 2^nd^ quintile |  |  | 0.00566 (0.0548) | 0.000688 (0.00665) | -0.00682 (0.0578) | -0.000795 (0.00674) |
| 1^st^ quintile (lowest 20%) |  |  | -0.00560 (0.0712) | -0.000680 (0.00866) | -0.0168 (0.0688) | -0.00196 (0.00802) |
| Married HH |  |  | -0.0899 (0.0712) | -0.0109 (0.00870) | -0.0760 (0.0687) | -0.00887 (0.00800) |
| HH education level (Reference: No formal education) |  |  |  |  |  |  |
| Some primary education |  |  | -0.282** (0.0997) | -0.0343** (0.0119) | -0.304** (0.0944) | -0.0355** (0.0110) |
| Primary education |  |  | -0.113+ (0.0672) | -0.0137+ (0.00831) | -0.171* (0.0669) | -0.0199* (0.00781) |
| Some secondary education |  |  | -0.0623 (0.0655) | -0.00757 (0.00812) | -0.104+ (0.0621) | -0.0122+ (0.00724) |
| Secondary education |  |  | 0.00423 (0.0890) | 0.000514 (0.0108) | -0.0766 (0.0810) | -0.00894 (0.00944) |
| Higher education |  |  | -0.0381 (0.0855) | -0.00462 (0.0104) | -0.108 (0.0847) | -0.0126 (0.00988) |
| Observations | 29420 | 29420 | 29303 | 29303 | 29303 | 29303 |
| Year Fixed Effects | Yes | Yes | Yes | Yes | Yes | Yes |
| District Fixed Effects | No | No | No | No | Yes | Yes |

+ p<.1, * p<.05, ** p<0.01, *** p<0.001 (Clustered standard errors in parentheses).

*Table 5-3. DiD analyses of the Bangladesh LFEP’s effect on incidence of CHE in treatment districts using* ***15% of THE*** *as a threshold*

|  | Equation 1 – No Controls | | Equation 2 – With Controls | | Equation 3 – Fixed Effects | |
| --- | --- | --- | --- | --- | --- | --- |
|  | Logit | MFX | Logit | MFX | Logit | MFX |
|  |  |  |  |  |  |  |
| Treatment district | 0.0590 (0.297) | 0.00561 (0.0283) | 0.0714 (0.299) | 0.00670 (0.0281) |  |  |
| DiD estimator | -1.299** (0.408) | -0.123** (0.0434) | -1.312** (0.415) | -0.123** (0.0436) | -1.078** (0.362) | -0.0964** (0.0326) |
| Number of chronically ill household members (Reference: 0 household members) |  |  |  |  |  |  |
| 1 household member |  |  | -0.246* (0.103) | -0.0231* (0.0103) | -0.256* (0.109) | -0.0229* (0.00965) |
| 2 household members |  |  | -0.270* (0.125) | -0.0254* (0.0122) | -0.253+ (0.139) | -0.0226+ (0.0124) |
| More than 2 household members |  |  | -0.176 (0.142) | -0.0165 (0.0138) | -0.134 (0.153) | -0.0120 (0.0137) |
| Number of household members |  |  | -0.0671*** (0.0198) | -0.00629*** (0.00181) | -0.0881*** (0.0137) | -0.00789*** (0.00121) |
| Rural |  |  | -0.150+ (0.0814) | -0.0140+ (0.00809) | -0.164* (0.0760) | -0.0146* (0.00679) |
| HH working for a livelihood |  |  | -0.403*** (0.0702) | -0.0378*** (0.00718) | -0.375*** (0.0705) | -0.0336*** (0.00642) |
| Female HH |  |  | -0.221* (0.0955) | -0.0207* (0.00894) | -0.238* (0.0932) | -0.0213* (0.00836) |
| Wealth quintile (Base: 3^rd^ quintile) |  |  |  |  |  |  |
| 5^th^ quintile (highest 20%) |  |  | -0.102 (0.0673) | -0.00959 (0.00620) | -0.0673 (0.0645) | -0.00602 (0.00577) |
| 4^th^ quintile |  |  | 0.0204 (0.0732) | 0.00191 (0.00691) | 0.0339 (0.0688) | 0.00304 (0.00617) |
| 2^nd^ quintile |  |  | -0.00726 (0.0567) | -0.000681 (0.00533) | -0.0216 (0.0594) | -0.00193 (0.00531) |
| 1^st^ quintile (lowest 20%) |  |  | -0.0508 (0.0904) | -0.00477 (0.00854) | -0.0564 (0.0845) | -0.00505 (0.00756) |
| Married HH |  |  | -0.194** (0.0739) | -0.0182* (0.00720) | -0.168* (0.0761) | -0.0150* (0.00680) |
| HH education level (Reference: No formal education) |  |  |  |  |  |  |
| Some primary education |  |  | -0.226+ (0.119) | -0.0212+ (0.0110) | -0.262* (0.112) | -0.0234* (0.00999) |
| Primary education |  |  | -0.0482 (0.0732) | -0.00452 (0.00691) | -0.133+ (0.0701) | -0.0119+ (0.00628) |
| Some secondary education |  |  | -0.101 (0.0693) | -0.00947 (0.00678) | -0.149* (0.0657) | -0.0133* (0.00587) |
| Secondary education |  |  | -0.0613 (0.0898) | -0.00575 (0.00856) | -0.165+ (0.0848) | -0.0147+ (0.00760) |
| Higher education |  |  | 0.0459 (0.0983) | 0.00431 (0.00917) | -0.0464 (0.0933) | -0.00415 (0.00835) |
| Observations | 29420 | 29420 | 29303 | 29303 | 29303 | 29303 |
| Year Fixed Effects | Yes | Yes | Yes | Yes | Yes | Yes |
| District Fixed Effects | No | No | No | No | Yes | Yes |

+ p<.1, * p<.05, ** p<0.01, *** p<0.001 (Clustered standard errors in parentheses

*Table 5-4. DiD analyses of the Bangladesh LFEP’s effect on incidence of CHE in treatment districts using* ***20% of TNFE*** *as a threshold*

|  | Equation 1 – No Controls | | Equation 2 – With Controls | | Equation 3 – Fixed Effects | |
| --- | --- | --- | --- | --- | --- | --- |
|  | Logit | MFX | Logit | MFX | Logit | MFX |
|  |  |  |  |  |  |  |
| Treatment district | -0.0563 (0.296) | -0.00725 (0.0379) | -0.0616 (0.306) | -0.00785 (0.0389) |  |  |
| DiD estimator | -0.724+ (0.375) | -0.0932+ (0.0505) | -0.726+ (0.385) | -0.0924+ (0.0514) | -0.379 (0.303) | -0.0465 (0.0372) |
| Number of chronically ill household members (Reference: 0 household members) |  |  |  |  |  |  |
| 1 household member |  |  | 0.105 (0.0881) | 0.0134 (0.0110) | 0.113 (0.0935) | 0.0139 (0.0115) |
| 2 household members |  |  | 0.180+ (0.107) | 0.0229+ (0.0132) | 0.202+ (0.118) | 0.0248+ (0.0145) |
| More than 2 household members |  |  | 0.277* (0.124) | 0.0353* (0.0149) | 0.325* (0.130) | 0.0399* (0.0160) |
| Number of household members |  |  | -0.0468** (0.0154) | -0.00596** (0.00196) | -0.0664*** (0.0123) | -0.00815*** (0.00150) |
| Rural |  |  | -0.0105 (0.0747) | -0.00134 (0.00954) | -0.0168 (0.0672) | -0.00206 (0.00825) |
| HH working for a livelihood |  |  | -0.384*** (0.0654) | -0.0489*** (0.00840) | -0.352*** (0.0658) | -0.0432*** (0.00805) |
| Female HH |  |  | -0.298*** (0.0695) | -0.0379*** (0.00886) | -0.308*** (0.0657) | -0.0378*** (0.00805) |
| Wealth quintile (Base: 3^rd^ quintile) |  |  |  |  |  |  |
| 5^th^ quintile (highest 20%) |  |  | -0.178** (0.0650) | -0.0226** (0.00801) | -0.151* (0.0614) | -0.0186* (0.00753) |
| 4^th^ quintile |  |  | -0.0588 (0.0738) | -0.00748 (0.00933) | -0.0515 (0.0713) | -0.00632 (0.00875) |
| 2^nd^ quintile |  |  | 0.0787 (0.0532) | 0.0100 (0.00665) | 0.0593 (0.0563) | 0.00728 (0.00691) |
| 1^st^ quintile (lowest 20%) |  |  | 0.182** (0.0686) | 0.0232** (0.00846) | 0.154* (0.0680) | 0.0189* (0.00833) |
| Married HH |  |  | -0.103 (0.0689) | -0.0132 (0.00897) | -0.0714 (0.0631) | -0.00877 (0.00775) |
| HH education level (Reference: No formal education) |  |  |  |  |  |  |
| Some primary education |  |  | -0.195* (0.0867) | -0.0248* (0.0109) | -0.220* (0.0859) | -0.0270* (0.0105) |
| Primary education |  |  | -0.109 (0.0677) | -0.0138 (0.00870) | -0.176** (0.0659) | -0.0216** (0.00808) |
| Some secondary education |  |  | -0.116+ (0.0605) | -0.0147+ (0.00793) | -0.163** (0.0579) | -0.0200** (0.00710) |
| Secondary education |  |  | -0.125 (0.0807) | -0.0159 (0.0105) | -0.218** (0.0770) | -0.0268** (0.00945) |
| Higher education |  |  | -0.159+ (0.0864) | -0.0202+ (0.0111) | -0.244** (0.0851) | -0.0300** (0.0104) |
| Observations | 29420 | 29420 | 29303 | 29303 | 29303 | 29303 |
| Year Fixed Effects | Yes | Yes | Yes | Yes | Yes | Yes |
| District Fixed Effects | No | No | No | No | Yes | Yes |

+ p<.1, * p<.05, ** p<0.01, *** p<0.001 (Clustered standard errors in parentheses).

*Table 5-5. DiD analyses of the Bangladesh LFEP’s effect on incidence of CHE in treatment districts using* ***25% of TNFE*** *as a threshold*

|  | Equation 1 – No Controls | | Equation 2 – With Controls | | Equation 3 – Fixed Effects | |
| --- | --- | --- | --- | --- | --- | --- |
|  | Logit | MFX | Logit | MFX | Logit | MFX |
|  |  |  |  |  |  |  |
| Treatment district | 0.0143 (0.322) | 0.00154 (0.0347) | 0.0156 (0.329) | 0.00166 (0.0351) |  |  |
| DiD estimator | -1.042** (0.405) | -0.112* (0.0473) | -1.045* (0.413) | -0.112* (0.0478) | -0.676* (0.340) | -0.0692* (0.0347) |
| Number of chronically ill household members (Reference: 0 household members) |  |  |  |  |  |  |
| 1 household member |  |  | -0.0708 (0.0957) | -0.00756 (0.0104) | -0.0693 (0.102) | -0.00709 (0.0104) |
| 2 household members |  |  | -0.0506 (0.124) | -0.00540 (0.0133) | -0.0286 (0.138) | -0.00293 (0.0141) |
| More than 2 household members |  |  | -0.0532 (0.128) | -0.00568 (0.0139) | 0.00426 (0.136) | 0.000436 (0.0139) |
| Number of household members |  |  | -0.0572** (0.0179) | -0.00610** (0.00192) | -0.0802*** (0.0144) | -0.00821*** (0.00147) |
| Rural |  |  | -0.0771 (0.0801) | -0.00822 (0.00875) | -0.0681 (0.0752) | -0.00696 (0.00769) |
| HH working for a livelihood |  |  | -0.361*** (0.0734) | -0.0386*** (0.00804) | -0.326*** (0.0732) | -0.0334*** (0.00750) |
| Female HH |  |  | -0.275*** (0.0806) | -0.0293*** (0.00862) | -0.289*** (0.0757) | -0.0296*** (0.00773) |
| Wealth quintile (Base: 3^rd^ quintile) |  |  |  |  |  |  |
| 5^th^ quintile (highest 20%) |  |  | -0.177* (0.0699) | -0.0189** (0.00720) | -0.149* (0.0658) | -0.0153* (0.00673) |
| 4^th^ quintile |  |  | 0.00770 (0.0802) | 0.000822 (0.00857) | 0.0171 (0.0773) | 0.00175 (0.00791) |
| 2^nd^ quintile |  |  | 0.0563 (0.0575) | 0.00601 (0.00601) | 0.0389 (0.0609) | 0.00398 (0.00623) |
| 1^st^ quintile (lowest 20%) |  |  | 0.0764 (0.0718) | 0.00815 (0.00750) | 0.0606 (0.0708) | 0.00620 (0.00724) |
| Married HH |  |  | -0.195** (0.0692) | -0.0208** (0.00775) | -0.164** (0.0622) | -0.0168** (0.00635) |
| HH education level (Reference: No formal education) |  |  |  |  |  |  |
| Some primary education |  |  | -0.266** (0.101) | -0.0284** (0.0107) | -0.299** (0.0980) | -0.0305** (0.0100) |
| Primary education |  |  | -0.112 (0.0724) | -0.0120 (0.00773) | -0.194** (0.0693) | -0.0199** (0.00708) |
| Some secondary education |  |  | -0.143* (0.0626) | -0.0153* (0.00706) | -0.196** (0.0601) | -0.0201** (0.00614) |
| Secondary education |  |  | -0.0847 (0.0822) | -0.00903 (0.00896) | -0.190* (0.0798) | -0.0195* (0.00816) |
| Higher education |  |  | -0.0974 (0.0896) | -0.0104 (0.00960) | -0.197* (0.0857) | -0.0202* (0.00877) |
| Observations | 29420 | 29420 | 29303 | 29303 | 29303 | 29303 |
| Year Fixed Effects | Yes | Yes | Yes | Yes | Yes | Yes |
| District Fixed Effects | No | No | No | No | Yes | Yes |

+ p<.1, * p<.05, ** p<0.01, *** p<0.001 (Clustered standard errors in parentheses).

*Table 5-6. DiD analyses of the Bangladesh LFEP’s effect on incidence of CHE in treatment districts* ***using 30% of TNFE*** *as a threshold*

|  | Equation 1 – No Controls | | Equation 2 – With Controls | | Equation 3 – Fixed Effects | |
| --- | --- | --- | --- | --- | --- | --- |
|  | Logit | MFX | Logit | MFX | Logit | MFX |
|  |  |  |  |  |  |  |
| Treatment district | 0.0212 (0.353) | 0.00199 (0.0332) | 0.0281 (0.356) | 0.00261 (0.0331) |  |  |
| DiD estimator | -1.263** (0.445) | -0.119* (0.0467) | -1.266** (0.454) | -0.118* (0.0470) | -0.874* (0.383) | -0.0773* (0.0339) |
| Number of chronically ill household members (Reference: 0 household members) |  |  |  |  |  |  |
| 1 household member |  |  | -0.260* (0.104) | -0.0241* (0.0102) | -0.267* (0.111) | -0.0236* (0.00974) |
| 2 household members |  |  | -0.314* (0.132) | -0.0291* (0.0129) | -0.296* (0.147) | -0.0262* (0.0130) |
| More than 2 household members |  |  | -0.414** (0.137) | -0.0385** (0.0146) | -0.351* (0.160) | -0.0310* (0.0141) |
| Number of household members |  |  | -0.0564** (0.0198) | -0.00524** (0.00182) | -0.0816*** (0.0148) | -0.00722*** (0.00129) |
| Rural |  |  | -0.143 (0.0875) | -0.0133 (0.00853) | -0.132 (0.0816) | -0.0117 (0.00721) |
| HH working for a livelihood |  |  | -0.377*** (0.0722) | -0.0350*** (0.00717) | -0.341*** (0.0719) | -0.0302*** (0.00640) |
| Female HH |  |  | -0.234** (0.0883) | -0.0217** (0.00816) | -0.250** (0.0808) | -0.0221** (0.00714) |
| Wealth quintile (Base: 3^rd^ quintile) |  |  |  |  |  |  |
| 5^th^ quintile (highest 20%) |  |  | -0.138+ (0.0705) | -0.0128* (0.00640) | -0.107+ (0.0638) | -0.00944+ (0.00564) |
| 4^th^ quintile |  |  | 0.0143 (0.0807) | 0.00133 (0.00751) | 0.0249 (0.0763) | 0.00220 (0.00676) |
| 2^nd^ quintile |  |  | 0.0442 (0.0586) | 0.00411 (0.00532) | 0.0275 (0.0615) | 0.00244 (0.00544) |
| 1^st^ quintile (lowest 20%) |  |  | 0.0153 (0.0840) | 0.00142 (0.00777) | 0.00483 (0.0802) | 0.000428 (0.00710) |
| Married HH |  |  | -0.188* (0.0767) | -0.0175* (0.00736) | -0.155* (0.0757) | -0.0137* (0.00667) |
| HH education level (Reference: No formal education) |  |  |  |  |  |  |
| Some primary education |  |  | -0.253* (0.106) | -0.0235* (0.00970) | -0.290** (0.101) | -0.0257** (0.00895) |
| Primary education |  |  | -0.0695 (0.0835) | -0.00645 (0.00780) | -0.161* (0.0815) | -0.0143* (0.00721) |
| Some secondary education |  |  | -0.141* (0.0712) | -0.0131+ (0.00701) | -0.194** (0.0707) | -0.0172** (0.00625) |
| Secondary education |  |  | -0.0753 (0.0895) | -0.00699 (0.00851) | -0.189* (0.0852) | -0.0168* (0.00754) |
| Higher education |  |  | -0.0616 (0.0974) | -0.00571 (0.00909) | -0.170+ (0.0893) | -0.0150+ (0.00790) |
| Observations | 29420 | 29420 | 29303 | 29303 | 29303 | 29303 |
| Year Fixed Effects | Yes | Yes | Yes | Yes | Yes | Yes |
| District Fixed Effects | No | No | No | No | Yes | Yes |

+ p<.1, * p<.05, ** p<0.01, *** p<0.001 (Clustered standard errors in parentheses).

*Table 5-7. DiD analyses of the Bangladesh LFEP’s effect on incidence of CHE in treatment district, using* ***40% of TNFE*** *as a threshold*

|  | Equation 1 – No Controls | | Equation 2 – With Controls | | Equation 3 – Fixed Effects | |
| --- | --- | --- | --- | --- | --- | --- |
|  | Logit | MFX | Logit | MFX | Logit | MFX |
|  |  |  |  |  |  |  |
| Treatment district | 0.0601 (0.400) | 0.00473 (0.0316) | 0.0847 (0.398) | 0.00653 (0.0309) |  |  |
| DiD estimator | -1.552** (0.529) | -0.122* (0.0479) | -1.567** (0.541) | -0.121* (0.0478) | -1.096* (0.480) | -0.0798* (0.0351) |
| Number of chronically ill household members (Reference: 0 household members) |  |  |  |  |  |  |
| 1 household member |  |  | -0.544*** (0.111) | -0.0420*** (0.00969) | -0.568*** (0.117) | -0.0413*** (0.00831) |
| 2 household members |  |  | -0.790*** (0.149) | -0.0609*** (0.0131) | -0.788*** (0.172) | -0.0574*** (0.0122) |
| More than 2 household members |  |  | -0.843*** (0.152) | -0.0650*** (0.0149) | -0.773*** (0.186) | -0.0562*** (0.0134) |
| Number of household members |  |  | -0.0369 (0.0236) | -0.00285 (0.00180) | -0.0634*** (0.0175) | -0.00461*** (0.00126) |
| Rural |  |  | -0.280** (0.0903) | -0.0216** (0.00768) | -0.278** (0.0903) | -0.0202** (0.00653) |
| HH working for a livelihood |  |  | -0.328*** (0.0799) | -0.0253*** (0.00643) | -0.286*** (0.0814) | -0.0208*** (0.00597) |
| Female HH |  |  | -0.230* (0.105) | -0.0178* (0.00798) | -0.246** (0.0953) | -0.0179** (0.00692) |
| Wealth quintile (Base: 3^rd^ quintile) |  |  |  |  |  |  |
| 5^th^ quintile (highest 20%) |  |  | -0.184* (0.0779) | -0.0142* (0.00601) | -0.148+ (0.0761) | -0.0108+ (0.00553) |
| 4^th^ quintile |  |  | 0.0125 (0.0881) | 0.000961 (0.00682) | 0.0245 (0.0845) | 0.00178 (0.00615) |
| 2^nd^ quintile |  |  | -0.0160 (0.0621) | -0.00123 (0.00482) | -0.0324 (0.0652) | -0.00236 (0.00474) |
| 1^st^ quintile (lowest 20%) |  |  | -0.0182 (0.108) | -0.00140 (0.00836) | -0.0167 (0.103) | -0.00121 (0.00749) |
| Married HH |  |  | -0.281** (0.0857) | -0.0216** (0.00697) | -0.244** (0.0847) | -0.0178** (0.00612) |
| HH education level (Reference: No formal education) |  |  |  |  |  |  |
| Some primary education |  |  | -0.291* (0.133) | -0.0224* (0.0102) | -0.331** (0.125) | -0.0241** (0.00906) |
| Primary education |  |  | -0.0558 (0.100) | -0.00430 (0.00774) | -0.167+ (0.0959) | -0.0122+ (0.00697) |
| Some secondary education |  |  | -0.156+ (0.0851) | -0.0120+ (0.00688) | -0.209* (0.0837) | -0.0152* (0.00607) |
| Secondary education |  |  | -0.0482 (0.0950) | -0.00372 (0.00740) | -0.174* (0.0857) | -0.0127* (0.00624) |
| Higher education |  |  | 0.0315 (0.123) | 0.00243 (0.00950) | -0.0874 (0.115) | -0.00636 (0.00838) |
| Observations | 29420 | 29420 | 29303 | 29303 | 29303 | 29303 |
| Year Fixed Effects | Yes | Yes | Yes | Yes | Yes | Yes |
| District Fixed Effects | No | No | No | No | Yes | Yes |

+ p<.1, * p<.05, ** p<0.01, *** p<0.001 (Clustered standard errors in parentheses).
