# Supplementary material for: Assessing the effects of disease-specific programs on health systems: An analysis of the Bangladesh Lymphatic Filariasis Elimination Program’s impacts on health service coverage and catastrophic health expenditure
Source: PLoS Negl Trop Dis. 2021 Nov 23;15(11):e0009894. doi: 10.1371/journal.pntd.0009894 (PMC8651132; doi:10.1371/journal.pntd.0009894)
Supplement: Attachment — Submitted filename: Koporc et al response to reviewers - 16 September 2021.pdf [file pntd.0009894.s007.pdf]

Kimberly M. Koporc, ScD, MPH, MBA
1661 La France Street, NE, Unit 414, Atlanta, GA 30307
+1-404-432-8030 ✻ kkoporc@gmail.com

September 16, 2021

Dr. Alberto Novaes Ramos Jr.
Associate Editor
PLOS Neglected Tropical Diseases

Dr. Francesca Tamarozzi
Deputy Editor
PLOS Neglected Tropical Diseases

RE: (PNTD-D-21-01100) - [EMID:1a3fb0ef7d450b85]

Dear Drs. Ramos and Tamarozzi,

As corresponding author, and on behalf of the co-authors, I wish to thank you for the positive and constructive feedback on our paper entitled, "Assessing the effects of disease-specific programs on health systems: An analysis of the Bangladesh Lymphatic Filariasis Elimination Program's impacts on health service coverage and catastrophic health expenditures." The following table provides a detailed description of our edits in response to each comment made by the reviewers.

No.	Reviewer	Comments	Edits
1	1	It would be my personal preference to have equations 6 & 9 listed along with equations 1-3 in the main text, since they are referred to throughout the results and tables.	Equations 6 and 9 were added to the main text (lines 303-314)
2	3	Are there concerns about ethical or regulatory requirements being met? Yes	We assume the reviewer meant to say "no" since there were no follow-up comments regarding ethical or regulatory requirements.
3	1	The numbers reported in the results feel abstract and it is hard to see whether there are meaningful (vs. statistically significant) differences. Is it possible to give greater interpretation of the results? For example, from the DID estimator can you report the OR and describe it in terms of increase in the dependent variable for each additional year that MDA was distributed during the 5 year period?	We agree that more explanation of what the numbers mean in the Results Section will facilitate reader comprehension. More explanations of the results are provided in lines 361-362, 374-375, and 412-416.

No.	Reviewer	Comments	Edits
4	1	…One important point that is lacking right now is an acknowledgment and discussion around the fact that the LF program was largely designed to sit outside the health system…	Agree. More discussion on this topic was added to the paragraph starting on line 61.
5	1	Line 34: the statement "except 5% of total household expenditures and 20% of total non-food expenditures" is not interpretable in the abstract as written; most readers will have no context for what these percentages mean. Suggest using more general language in the abstract (same note for the author summary)	Agree. Percentages were removed from the abstract as suggested. The percentages were not originally included in the author summary. Therefore, no changes were made to the author summary
6	1	Line 37: Is this referring to positive associations between LF program interventions? If so, please specify "LF"	"Lymphatic Filariasis Elimination Program's" was inserted in line 38.
7	1	Line 103 - 112: is there a reason why DEC is omitted?	DEC was added (lines 108-109). This was an important omission. Thank you to the reviewer for catching it.
8	1	Line 161: this sentence would be easier to interpret if you said, "...was identified by plotting the global positioning system coordinates of the PSU on a 2015..."	Agree. Edit made to lines 166-167.
9	1	Table 3: Can you add the variable names in parentheses to this table so that it matches what's written in Eqns 1-3?	There were a few typos in Table 3, which regretfully may have caused confusion. These typos were corrected. The variables in Table 3 now match what is written in equations 1-3 for health service coverage rates
10	1	Lines 264 - 276: Greater explanation of subscripts is needed for these equations	Definitions of the estimating equations' terms are already included in the text box in Supplement 3 (last page). We added "…and definitions of the terms are presented in Supplement 3" to the main text (Line 271) and deleted line 293.
11	1	Line 435: Should this instead read, "One possible reason for the increase in FINANCIAL PROTECTION is positive interactions with the health staff..." or something to that effect? This statement seems to be referring to the significant CHE outcome.	Agree. Edit made to line 460

No.	Reviewer	Comments	Edits
12	2	Line 33 - consider removing 'the' before incidence	Agree. Edit made to line 33
13	2	Lines 134 and 135 - consider using abbreviations instead (IEC, MMDP and M&E) instead of writing out in full. Already done in lines 110, 111 and 112.	Agree. Edit made to lines 140-141
14	2	Lines 184 and 185 - consider putting FP after the first family planning and use FP afterwards instead of at the end of the sentence.	Agree. Edit made to lines 190-191
15	2	Line 453 - consider adding 'of' before OOPs	Agree. Edit made to line 478.
16	1	Summary and general comments	Noted. Thank you
17	2	Summary and general comments	This reviewer raises important issues, some of which are mentioned in the limitations section. Therefore, making the peer-review history publicly available would be beneficial.<br><br>Regarding the comment "We need to consider vertical versus horizontal equity because within each of the quintiles we use there could be differences and thus the assumption we make that each quintile we identify is homogenous could be flawed," we used an asset index for 16 different strata of HIES data to account for economic and cultural differences and urban and rural environments (lines 244-259).
18	3	Summary and general comments	Reviewer 1 also requested expanding the discussion on integration. See comment number 4 of this table.<br><br>The tracer indicators used in the analysis were established by World Health Organization and World Bank and are widely used in the literature. We address the selection of indicators in lines 124-128 and 505-512. We, and it seems Reviewer 3, agree with the general comments made by Reviewer 2 that more work needs to be done to identify the best tracer indicators to use.

No.	Reviewer	Comments	Edits
			Reviewer 3 raises some of the same important issues as Reviewer 2.
19	Comments in attachment	Line 28, Line 166 - add "year"	Edits made. Authors also add "years" in lines 206 and 397.
20		Add percentages to first rows of Tables 4 and 6	This suggestion may facilitate reader comprehension. Edits made
21		"Won't it be better if the table comes first before the explanation. I am just suggesting because reader appreciate that more to this format."	This suggested edit was not made. It is standard practice to refer to tables and figures in the text before presenting them in the manuscript.
22	Additional edits made by authors	Included middle initial of Dr. Deborah A. McFarland	
23		Lines 112-113 – Edited sentence to make it concise	
24		Line 370 – Changed "Table 6" to "Table 5"	

As requested, two versions of the revised manuscript are included: one with either highlights or tracked changes denoting where the text has been changed; the other a clean version. Thank you for considering our paper for publication in *PLOS Neglected Tropical Diseases.*

Sincerely,

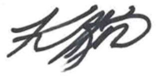

Kimberly M. Koporc, ScD, MPH, MBA
